# Motivators and barriers of tamoxifen use as risk-reducing medication amongst women at increased breast cancer risk: a systematic literature review

**DOI:** 10.1186/s13053-017-0075-8

**Published:** 2017-09-20

**Authors:** B. Meiser, W. K. T. Wong, M. Peate, C. Julian-Reynier, J. Kirk, G. Mitchell

**Affiliations:** 1 Prince of Wales Clinical School, UNSW, Level 4, Lowy Cancer Research Centre C25, Sydney, NSW 2052 Australia; 2 School of Social Sciences and Prince of Wales Clinical School, UNSW Sydney, Kensington, NSW 2052 Australia; 3 Department of Obstetrics and Oncology, Royal Women’s Hospital, University of Melbourne, Melbourne, VIC 3052 Australia; 4 Institut Paoli-Calmettes, Marseille, France; 5 Familial Cancer Service, Westmead Hospital, Hawkesbury Road, Westmead, NSW 2145 Australia; 6 Westmead Millennium Institute for Medical Research at the University of Sydney, PO Box 412, Westmead, NSW 2145 Australia; 7 Sir Peter MacCallum Dept of Oncology, University of Melbourne, Parkville, VIC 3010 Australia; 8 Familial Cancer Centre, Peter MacCallum Cancer Centre, Melbourne, VIC 8006 Australia

**Keywords:** Tamoxifen, Prevention, Risk-reducing medication, Breast cancer, High risk

## Abstract

**Background:**

Selective estrogen receptor modulators, such as tamoxifen, reduce breast cancer risk by up to 50% in women at increased risk for breast cancer. Despite tamoxifen’s well-established efficacy, many studies show that most women are not taking up tamoxifen. This systematic literature review aimed to identify the motivators and barriers to tamoxifen use ‘s amongst high-risk women.

**Methods:**

Using MEDLINE, PsycINFO, and Embase plus reviewing reference lists of relevant articles published between 1995 and 2016, 31 studies (published in 35 articles) were identified, which addressed high-risk women’s decisions about risk-reducing medication to prevent breast cancer and were peer-reviewed primary clinical studies.

**Results:**

A range of factors were identified as motivators of, and barriers to, tamoxifen uptake including: perceived risk, breast-cancer-related anxiety, health professional recommendation, perceived drug effectiveness, concerns about side-effects, knowledge and access to information about side-effects, beliefs about the role of risk-reducing medication, provision of a biomarker, preference for other forms of breast cancer risk reduction, previous treatment experience, concerns about randomization in clinical trial protocols and finally altruism.

**Conclusions:**

Results indicate that the decision for high-risk women regarding tamoxifen use or non-use as a risk-reducing medication is not straightforward. Support of women making this decision is essential and needs to encompass the full range of factors, both informational and psychological.

## Background

The use of selective estrogen receptor modulators (SERMs) as a risk-reducing strategy for women at moderate to high risk for hereditary breast cancer, including women with a *BRCA1* or *BRCA2* mutation, has been the focus of a number of research studies e.g., [1, 2]. Study findings have shown that the use of these agents (e.g., tamoxifen) can reduce the risk of developing estrogen receptor positive breast cancer by up to 50% [3]. However, similar to any other medicine, tamoxifen has known side-effects and has been found to be associated with a number of increased health risks [4]. For example, the drug is linked to endometrial cancer in post-menopausal women, pulmonary embolism, stroke, thromboembolic events, cataracts, menopausal symptoms such as hot flashes and night sweats, vaginal discharge and sexual problems [3]. Accordingly the U.S. Preventive Services Task Force and American Society of Clinical Oncology guidelines recommend that women at increased risk and their clinicians engage in shared, informed decision-making about these medications [5, 6].

While findings from some studies suggest that tamoxifen is an acceptable risk-reducing strategy among women with high breast cancer risk [7], the actual reported uptake of tamoxifen by such women is low e.g., [8–12]. In a recent meta-analysis of uptake of therapeutic agents to prevent breast cancer among women at increased risk, Smith et al. [13] found that only 16.3% (95% CI, 13.6–19.0) of women took up risk-reducing medication. Moreover, findings from some research studies suggest that the availability of information to women about tamoxifen can impact on their decision-making process [14–16].

The proven efficacy of tamoxifen as risk-reducing medication is in striking contrast with the low rate of uptake by women who might benefit from it. Therefore, it is important to understand the motivators and barriers to tamoxifen use that have been identified in peer-reviewed publications. This review will provide clarity about the issues that are important to high-risk women with respect to tamoxifen as a risk-reducing strategy. This current systematic review extends the review of the factors associated with uptake of risk-reducing medication by Smith et al. [13], who performed a meta-analysis of risk-reducing medication uptake as well as a systematic review of the motivators and barriers associated with actual uptake of risk-reducing medication (total of 21 studies). The number of studies included in the current review of motivators and barriers is larger (31 studies), because our review includes studies assessing the motivators and barriers towards both actual and hypothetical (intended) uptake of risk-reducing medication. It was decided to include both types of studies to capture a wide range of motivators and barriers to the use of tamoxifen as a risk-reducing strategy, and to provide the basis for the development of novel and comprehensive strategies relating to its use.

## Methodology

### Search strategy

The literature review procedure took place as follows. The electronic databases Embase, Ovid MEDLINE and PsycINFO were searched from 1995 (the year when studies on the efficacy of tamoxifen as a risk-reducing medication in high-risk women started to get published) to December 2016. Articles were included if they: assessed motivators and/or barriers for the use of SERM in women at increased risk for breast cancer; and were peer-reviewed primary clinical studies and published in the English language. Articles were excluded if they were: review articles, conference abstracts, editorials/commentaries, recommendations, or case studies or if they included women with a personal diagnosis of breast cancer. In each database, searches for the disease types terms (‘breast neoplasms’, ‘BRCA1’, ‘BRCA2’, and ‘hereditary breast cancer’), the treatment modalities or intervention of interest (‘chemoprevention’, ‘tamoxifen’, ‘prevention’, ‘risk reduction’, ‘risk-reducing medication’, ‘RRM’, ‘selective estrogen receptor modulators’, and ‘SERMS), and the outcomes of interest (‘barriers’, ‘incentives’, ‘attitudes’, ‘motivation’, ‘decision making’, ‘decision-making’, ‘communication barriers’, ‘anxiety’, and ‘health behavior’) were conducted by MP and BM and combined, with duplicates removed. To augment the electronic search, reference lists of included studies were examined manually to identify additional relevant studies by MP and BM. Both authors agreed upon articles for inclusion. The review was guided by the Preferred Reporting Items for Systematic Reviews and Meta-Analyses (PRISMA) Statement [17].

## Results

**Fig. 1:**
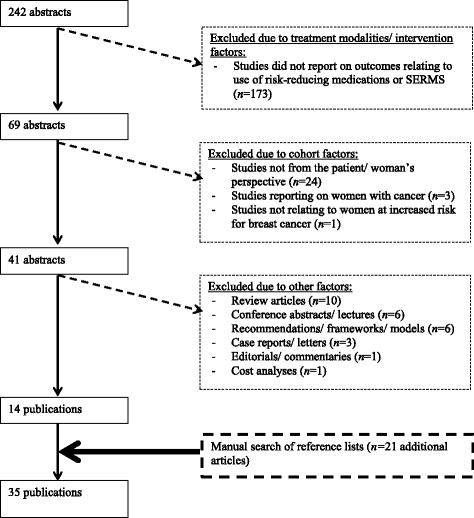
PRISMA flow diagram displaying articles included and excluded

Figure 1 shows a PRISMA flow diagram displaying the number of included and excluded articles at different stages of the literature search, including the reasons for exclusion. Of the 242 abstracts identified, 173 were excluded because they did not report on outcomes relating to use of risk-reducing medication; 28 were excluded due to cohort factors and 27 due to other factors, leaving 14 articles that met the eligibility criteria. A manual search identified an additional 21 articles, bringing up the total number of articles meeting inclusion criteria to 35, the details of which are summarized in Table 1. These 35 articles reported on 31 different studies, and the majority of studies (17) were undertaken in the United States; while five were undertaken in Canada; two each in the United Kingdom, Italy and Australia; one each in France and Germany; and one study involved participants in Canada, France, and the United Kingdom. Eight studies used a qualitative design and 23 a quantitative design. Of the quantitative studies, 11 employed a cross-sectional and 12 a prospective design. Nine studies recruited women through familial cancer or high-risk breast cancer clinics or a cancer genetics research program [1, 2, 7, 16, 18–22], while the remaining studies recruited through a variety of clinic and community settings.

**Table 1:** Studies reviewed

Author and country	Population	Study design
Altschuler, A. et al. [23], United States	51 women at increased risk according to Gail model* and eligible for chemoprevention trial	Semi-structured in-depth in-person interviews
Bober, S. L., et al. [24], United States	129 increased–risk women according to Gail model* following cancer risk counselling	Self-administered questionnaires and telephone interviews at 2 and 4 months post-counselling
Cyrus-David, M. et al. [39], United States	26 women at increased risk	Qualitative study reporting focus group data
Dillard, A.J., et al. [15] & Dillard, A. J., et al. [32], United States	632 women at increased risk based on Gail model* score risk who were administered a decision aid	Self-administered questionnaires before and after reading decision aid
Donnelly, L.S. [1], United Kingdom	30 women at high risk (≥17% lifetime risk according to Tyrer-Cuzick model) ascertained through a high-risk clinic	Semi-structured interviews
Fagerlin, A., et al. [9] & Fagerlin, A., et al. [29], United States	663 women at increased risk according to Gail model* recruited through large health maintenance organizations	Self-administered questionnaires at baseline, post-decision aid and 3 months post-decision aid
Fallowfield, L., et al. [11], United Kingdom	488 women at high familial risk considering entry into chemoprevention trials	Self-administered questionnaires every 6 months
Goldenberg, V. K. [18], United States	99 women at increased risk according to Gail model* attending a clinic for high-risk breast cancer who received fine needle aspiration results designed to evaluate breast cancer risk	Women were followed up regarding the impact of their cytology results on decision-making pertaining to the use of tamoxifen for breast cancer chemoprevention
Heisey, R., et al. [8], Canada	27 women at increased risk	Semi-structured in-person interviews
Julian-Reynier, C, et al. [19], Canada, United Kingdom and France	355 women attending genetic clinics in Marseille, (*n* = 141), Manchester (*n* = 130) and Quebec (*n* = 84)	Self-administered questionnaire before consultation, consultant completed questionnaire after consultation
Julian-Reynier, C., et al. [25], France	246 carriers and non-carriers who were tested for BRCA1/2 mutations 5 years prior	Six self-administered questionnaires over 5 years
Kinney, AY et al. [35–37], United States	175 women at increased risk for breast cancer who attended an information session about a breast cancer prevention trial and discussed participation with their physician	Self-administered questionnaire
Loehberg, C.R., et al. [40], Germany	199 women at increased risk according to Tyrer–Cuzick model who were eligible for but declined participation in a chemoprevention trial	Self-administered questionnaire
Matloff, E.T., et al. [30], United States	48 cancer-free women with a first-degree relative with breast cancer	Self-administered questionnaires at baseline, 1 and 6 months, participants randomized to a genetic counseling intervention or control
McKay, A., et al. [14], Canada	51 women at high risk of breast cancer according to Gail model* and seen by surgeons	Self-administered questionnaire
McKinnon, W. [41], Canada	34 BRCA1/2 mutation carriers invited to a one-day retreat	Self-administered questionnaires at baseline and 6 months
Meiser, B. [26], Australia	371 women from multiple-case breast cancer families	Self-administered questionnaire
Melnikow, J., et al. [12], United States	255 women at increased risk according to Gail model* recruited through university medical center and at community sites	Qualitative and quantitative in-person interview
Metcalfe, K. A., et al. [2], Canada	81 BRCA1/2 mutation carriers who were identified through the records of two cancer genetics clinics	Mailed, self-administered questionnaire
Muir, A. [20], Australia	35 women who had attended a familial cancer clinic and were eligible for a chemoprevention trial were contacted 6 months - 7 years after clinic attendance	Structured telephone interview
Paterniti, D.A. et al. [27], United States	27 high-risk women (African-American, White, and Latina) sampled through community organizations	3 separate focus group interviews with African- American, White, and Latina women plus post-focus group self-administered questionnaire
Port, E.R. [38], United States	43 at increased risk eligible to take tamoxifen for primary prevention*	Completion of baseline self-administered questionnaire, followed by educational sessions and literature on tamoxifen use, followed by questionnaire and telephone interview
Razzaboni, E., et al. [10], Italy	471 women at increased risk eligible for chemoprevention trial	Semi-structured interviews
Rondanina, G. et al. [42], Italy	457 women on hormone replacement therapy who were invited to participate in a low-dosage tamoxifen trial	Self-administered questionnaire
Salant, T. et al. [21], United States	33 high-risk women (75% African-American) recruited through a high-risk breast cancer clinic	Semi-structured interviews
Schwartz, M. D., et al. [7], United States	465 women who had genetic counselling and testing through clinical genetics research program	Choice of mailed self-administered survey or telephone survey
Stacey, D., et al. [16], United States	97 high-risk women with a 1.66% or greater five year, referred a high-risk breast assessment clinic	Mailed, self-administered questionnaire
Taylor, R et al. [31], Canada	89 women at high risk who were evaluated for a breast lump at a referral center	Telephone survey
Tchou, J. et al. [43], United States	137 women attending a breast center who were offered tamoxifen	Review of medical files
Tjia, J., et al. [34], United States	457 community dwelling women aged 60–65 years old who were potentially eligible for breast cancer chemoprevention according to Gail model*	Mailed, self-administered survey
Underhill, M.L. [22], United States	21 women at high risk recruited from a high-risk breast program	In-depth interviews

Legend: * > 1.7% 5-year breast cancer risk, entry criterion for National Surgical Adjuvant Breast and Bowel Project trial

This review identified a range of factors acting as motivators and barriers to tamoxifen use.

### Perceived risk

Women who believed they were likely to develop breast cancer reported being more likely to take tamoxifen to reduce their risk [8, 15, 19, 23–28]. Conversely studies have shown that women who believed their breast cancer risk to be low were less likely to be interested in taking the drug [12, 21, 23, 29–31].

### Breast cancer-related anxiety/worry

Women who were worried about breast cancer were more likely to be interested in, or take up, risk-reducing medication for breast cancer than women who were not [23, 24, 29, 32, 33]. Of note, worry about breast cancer was found to be more strongly associated with an interest in risk-reducing medication than perceived risk of breast cancer [34]. Given anxiety has been described as an “action emotion” [32], p.17, it has been argued that women were more likely to take up tamoxifen as a way of managing this emotion.

### Health professional recommendation

Women who reported that their doctor recommended risk-reducing medication were more likely to take it up [24] or enroll in a breast cancer prevention trial [35–37]. Conversely a lack of health professional recommendation can be a reason for not using it [2]. Moreover, based on their findings, Port et al. [38] asserted that “negatively biased physician presentations” (p.583) can encourage the decision to not take tamoxifen.

### Perceived drug effectiveness

The decision to take tamoxifen is influenced by the perceived effectiveness of the proposed treatment. Study findings have shown that women would consider taking tamoxifen if it were shown that it could definitively prevent breast cancer [8, 26]. Conversely one study [29] indicated that reluctance of some women to take tamoxifen was based on their views that the drug would not substantially reduce the risk of breast cancer. In another study, women perceived a lack of sufficient data to support the effectiveness of tamoxifen [31]. Women’s perceptions were that there was no guarantee that tamoxifen would prevent their own breast cancer, given it reduced risk by 50% only [21].

### Concerns about side-effects

Many of the studies identified found that concerns about tamoxifen-related side-effects were a barrier to the use of risk-reducing medication or influenced women’s decision to not use the drug [1, 2, 8, 12, 22, 28, 38–43] or to participate in tamoxifen prevention trials [23]. For example, Paterniti et al. [27] found that women in their study were less willing to use tamoxifen for breast cancer risk reduction due to concerns about its side-effects, given the availability of other options such as diet, exercise and regular screening. Indeed, taking medicines for risk reduction was viewed as ‘unnatural’ and there was a perception that it would interfere with body integrity [8].

### Knowledge and access to information about side-effects

Fagerlin et al. [9] found that women who had poor knowledge of tamoxifen’s risks were more likely to be interested in taking it. Conversely, studies showed that women who were clearly informed of the benefits and risks of tamoxifen were less likely to take up tamoxifen as a risk-reducing medication [9, 12, 14, 27]. These findings indicate that women who know less about the potential side-effects of tamoxifen may be less focused on such side-effects and only consider its risk-reducing effects.

### Beliefs about the role of risk-reducing medication

Studies also documented that women did not want to take medication on a regular basis [38]; also, women did not want to take tamoxifen because it is a cancer treatment drug, and thus taking tamoxifen served as a daily reminder of their cancer risk [1]. In a similar vein, Salant et al. [21] found that many women felt that a medication was to be taken only after a problem had arisen, as opposed to preventing it [8].

### Provision of a biomarker

In a study by Goldenberg et al. [18], women at increased risk who received fine needle aspiration results to evaluate their breast cancer risk were followed up regarding the impact of their cytology results on decision-making about tamoxifen use. Results show that 50% of women with atypia elected to take tamoxifen, compared to 7% women with borderline aplasia, suggesting that the provision of a biomarker of individual breast cancer risk can affect the motivation to take tamoxifen [18].

### Preference for other forms of breast cancer risk reduction

Metcalfe et al. [2] found that more women preferred either risk-reducing mastectomy or bilateral oophorectomy than to take tamoxifen. For example, they found that women were five times more likely to opt for risk-reducing bilateral oophorectomy than to use risk-reducing mediation to reduce breast cancer risk. Moreover, Metcalfe et al. [2] suggest that other risk-reducing measures (e.g. risk-reducing mastectomy) were preferred, because women considered them to be more permanent risk-reducing strategies when compared with tamoxifen.

### Previous treatment experience

Previous treatment experiences may also affect willingness to use tamoxifen. For example, Julian-Reynier et al. [19] found that women who had undergone risk-reducing bilateral oophorectomy were less interested in risk-reducing medication than women who had not. This latter finding is perhaps not surprising given risk-reducing bilateral oophorectomy in previously premenopausal women has been shown to reduce breast cancer risk by 50% [44], and it is unclear whether there are additional benefits of tamoxifen in this setting. Previous treatment experience can also occur vicariously. For example, results indicate that women who observed the negative effects of tamoxifen as a treatment for cancer in other women were less likely to consider the drug as a risk-reducing medication [1, 22].

### Clinical trials context

Clinical trial protocols have reported that randomization reduces women’s willingness to participate in trials [45]; despite this, women who accepted drug randomization as part of the protocol were more in favor of risk-reducing medication than those who did not [19]. Also, Muir et al. [20] found that one of the most cited reason for not participating in trials of risk-reducing medication was the view that drug treatment should be the last option for prevention purposes.

### Altruism

Despite their concerns about side-effects related to the use of medication for reducing breast cancer risk, women participating in, and eligible for, tamoxifen trials reported that they would use the medication to help others by being involved in endeavours that would advance understanding and prevention of breast cancer [8, 23].

## Discussion

This article extends previous reviews of uptake of risk-reducing medication [13, 28] by examining a broad range of motivators and barriers associated with use of, and decision-making about, risk-reducing medication in women at increased risk for breast cancer. Compared to the Smith et al. study [13], an additional 10 studies specifically examining motivators and barriers were included. Our systematic review identified several factors influencing women’s decisions about tamoxifen use that are similar to those that influence women’s decisions about other risk-reducing strategies (e.g. risk-reducing surgery), including perceived risk and breast cancer-related anxiety. For example, previous research findings showed that women opting for risk-reducing surgery may be motivated by high levels of anxiety about breast/ovarian cancer [46] and/or high levels of perceived risk [26]. Our review showed that perceived risk and breast cancer anxiety motivated women to consider or take up risk-reducing medication, while low level of risk and anxiety acted as barriers. Thus, in accordance with well-known conceptual models of health behaviors, including the Health Belief Model, the Transactional Model of Stress and Coping and Self-Regulation Theory, perceived risk or susceptibility appears to be a key dimension underlying uptake of preventative behaviors [47–49], including uptake of medication to reduce the risk of breast cancer.

The Health Belief Model includes one construct, cues to action, to predict health-promoting behaviors; such cues may be either internal (e.g. symptom) or external (e.g. physician’s recommendation) [50]. Illustrating an internal cue to action, this review identified a study by Goldenberg et al. [18], which demonstrated that the provision of a biomarker can affect the motivation to take tamoxifen. In a study illustrating the importance of an external cue to action, Bober et al. [24] reported that women whose doctor recommended the use of risk-reducing medication were more likely to take up this treatment as a way to decrease the likelihood of developing breast cancer, underscoring the findings from numerous studies that document the key role of physician recommendation in influencing screening and preventative behaviors [46].

Not surprisingly, this review showed that concerns about side-effects were a key barrier to consideration and use of tamoxifen [10, 20]. Interestingly, it appeared that some side-effects are more acceptable than others. For example, Metcalfe et al. [2] found that women were more negative towards tamoxifen-related side-effects than those related to other risk-reducing measures (e.g. risk-reducing bilateral oophorectomy). Metcalfe et al. [2] discussed the differences between these two preventative strategies that may influence women’s preferences. In particular, risk-reducing bilateral oophorectomy also confers protection against ovarian cancer, a strategy relevant to *BRCA1/2* carriers and other women with hereditary breast/ovarian cancer; oophorectomy has side-effects for premenopausal women, such as loss of fertility and sudden onset of menopause; and tamoxifen has been shown to be associated with an increased risk of endometrial cancer, deep vein thrombosis and pulmonary embolus. These authors argue that although risk-reducing bilateral oophorectomy and tamoxifen have similar preventative effectiveness, women seem to place more weight on the side-effects of tamoxifen than those of oophorectomy [2].

Thus, the use of risk-reducing medication for women with a high breast cancer risk is a complex decision and is strongly influenced by personal perceptions and values. This review suggests that the low rates of uptake of tamoxifen may be partly addressed by providing women with specific information about some of the motivating factors identified. First, women should be provided with accurate information regarding their breast cancer risk, which in turn is likely to motivate consideration of tamoxifen use. Genetic risk information should be accompanied by information on the well-established efficacy of risk-reducing medication as well careful description of the documented side-effects related to the medication with respect to the actual magnitude of the potential cancer risk reduction. Women should also be provided with information on the time duration to achieve the effect. The recommended duration of tamoxifen (five years) for risk reduction is based on data on contralateral breast cancers among women who were treated for primary breast cancer. However, the optimum duration of tamoxifen for treatment of breast cancer and for risk reduction are not necessarily the same. Due to the risk of side-effects, the shortest intervention is preferable. In a case-control study, Gronwald et al. [51] observed that 1-year of tamoxifen use was sufficient to achieve a reduction in the risk of breast cancer. These authors suggest that a randomized controlled trial of 1-year of tamoxifen versus placebo in *BRCA1* and *BRCA2* mutation carriers is warranted.

The decision to take or not take tamoxifen is a preference-sensitive decision, and as such, decisions are not always a straightforward balancing of absolute risks and benefits. Decisions aids are likely to be beneficial to facilitate decision-making about tamoxifen as risk-reducing medication [52]. Women should be encouraged to explore their own needs, perceived risks and how the treatment might impact on their everyday lives, to enable them to make an informed decision about the use of risk-reducing medication. Given the documented impact of physician recommendation on women’s decisions about tamoxifen use [24, 35], it is equally important to ensure optimal education of physicians around the role of tamoxifen as prevention strategy and to train physicians in how to best facilitate women’s informed decisions. Also physicians should be encouraged to discuss risk-reducing medication with all increased-risk women, which may be achieved through clinical practice guidelines.

Prior to concluding, the limitations of this review should be noted. Due to the small number of studies assessing particular factors, it was not possible to synthesize the data in a meta-analysis. The review included studies assessing both actual and hypothetical uptake of risk-reducing medication, and the motivators and barriers associated with hypothetical uptake may be different from those related to actual uptake. The relatively narrow search items led to somewhat limited sensitivity achieved in this review, with 14/35 (40%) of eligible articles identified directly by the electronic search, with the remainder identified through manual searches of reference lists. Due to the inclusion criteria, articles published in languages other than English were not included. The review was further limited by the low number of studies that were conducted in countries outside the US and Canada. Many of the studies included in the review were published some time ago, and more data have become available since to support the efficacy of tamoxifen [53]. It is possible that women’s preferences have changed as a result of recent evidence, and the replication of studies such as the one by Metcalfe et al. [2] would be of interest.

## Conclusion

As the decision about whether or not to take tamoxifen is a complex one with a range of influencing factors, women may be best supported in their decision to commence tamoxifen and continue with its use as a prevention strategy by a multidisciplinary team that can address the range of factors. Furthermore it needs to be acknowledged that the decision may require several contacts with the team before a woman decides that tamoxifen may be a suitable breast cancer prevention strategy for her. Once there is a groundswell of use of tamoxifen, positive reviews of tamoxifen posted by women using it on social media and support forums and more experience of physicians in initiating and prescribing tamoxifen as a prevention strategy may lead more women to consider it a reasonable strategy.

## Abbreviations

PRISMA: Preferred Reporting Items for Systematic Reviews and Meta-Analyses
SERM: Selective estrogen receptor modulator
